# NRF2 regulates lipid droplet dynamics to prevent lipotoxicity

**DOI:** 10.1016/j.isci.2025.112925

**Published:** 2025-06-18

**Authors:** Christopher T. Prevost, William B. Gansereit, David F. Kashatus

**Affiliations:** 1 Department of Biochemistry and Molecular Genetics, University of Virginia Health System, Charlottesville, VA 22908, USA; 2 Department of Microbiology, Immunology, and Cancer Biology, University of Virginia Health System, Charlottesville, VA 22908, USA

**Keywords:** Biological sciences, Biochemistry, Cell biology

## Abstract

Lipid droplets (LDs) are dynamic organelles comprising a neutral lipid core encapsulated by a phospholipid monolayer. LD structure and function are influenced by a variety of intrinsic and extrinsic signals, and cells alter LD content and distribution to adapt to their environment. Here, we show that LD content increases in response to stabilization of the transcription factor NRF2 under conditions of lipotoxic stress. Notably, NRF2 activity leads to increased expression of the G0S2, a protein that inhibits ATGL, the enzyme responsible for degradation of triacylglycerol and the release of fatty acids from LDs. Importantly, stabilization of NRF2 in the absence of stress is sufficient to increase LD content, and inhibition of ATGL partially rescues the impact of NRF2 deletion on stress-induced ferroptosis. These data support a model in which stress-induced NRF2 stabilization protects cells against lipotoxicity in part through the sequestration of fatty acids in lipid droplets.

## Highlights

• NRF2 expression promotes increased lipid droplet content
• NRF2-dependent G0S2 expression inhibits lipid droplet degradation, promoting lipid storage
• Polyunsaturated fatty acids promote NRF2 stability
• Inhibition of lipid droplet degradation protects cells against lipotoxicity

## Graphical abstract

**Figure:**
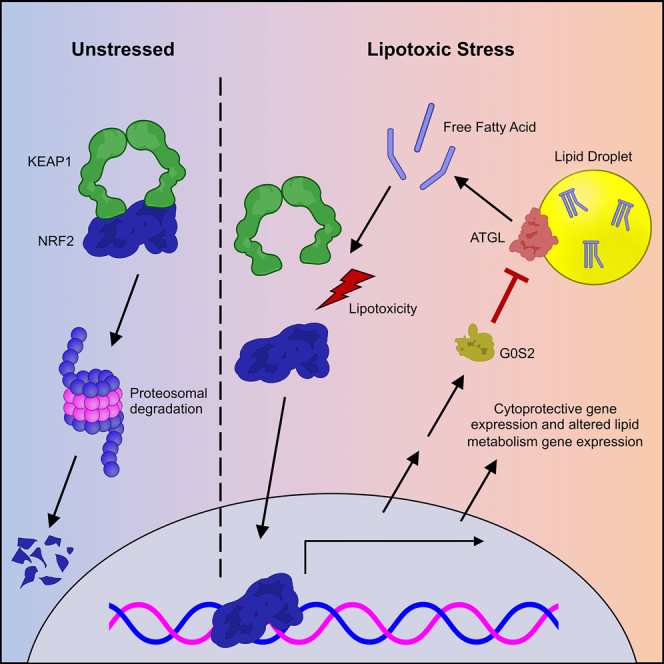

## Introduction

When cells encounter stress, molecular sensors are activated and initiate a series of downstream signaling cascades that alter cellular processes to mitigate stress-induced damage. Part of this coordinated stress response are changes to the structure and function of cellular organelles, including lipid droplets (LDs). These spherical organelles comprise a neutral lipid core that is sequestered from the cytoplasm by a phospholipid monolayer.^1^ LDs are highly dynamic and, in addition to energy storage, they have been shown to influence processes such as cell signaling, membrane biogenesis, lipotoxicity,^2^ and transcription.^3^ LD content increases or decreases based on metabolic needs or environmental conditions. For example, during conditions of hypoxic stress, the transcription factor hypoxia-inducible factor 1 (HIF-1α) is stabilized,^4^ driving the expression of genes involved in LD regulation, notably HILPDA (also known as HIG2) which can inhibit triacylglycerol (TAG) degradation and increase storage of fatty acids as TAGs.^5^^,^^6^ Additionally, cells under nutrient stress increase autophagy, and the fatty acids released through this process are esterified, and subsequently stored as TAG inside LDs.^7^^,^^8^

Although LDs are increasingly recognized as organelles that play substantial roles in both normal and pathological physiology, the regulation of the machinery that controls their dynamic behavior remains incompletely understood. For example, although LDs respond dynamically to lipotoxic stress,^9^ the molecular mechanisms through which lipotoxic stress sensors communicate with the LD regulation machinery are not well defined. To gain a better understanding of the relationship between LDs and the response to lipotoxic stress, we sought to investigate a the transcription factor NRF2 (NFE2 like bZIP transcription factor 2), which is a member of the basic leucine zipper family of transcription factors,^10^ crucial for the ability of cells to cope with electrophilic, xenobiotic, and oxidative stress.^11^ NRF2 is known to drive the expression of genes that neutralize lipotoxic stress,^12^ but there is limited research on its relationship to LDs. Notably, it has been observed that DGAT1, a protein that mediates TAG synthesis, protects melanoma cells against lipid peroxidation and apoptosis, and its inhibition increases the expression of genes transcribed by NRF2 activity.^13^ Additionally, NRF2 was observed to be vital for adipogenesis and accumulation of white adipose tissue in mice,^14^ alter lipogenic gene expression,^15^ and was recently found to be vital for the uptake of lipids in β-cells through induction of the fatty acid transporter CD36.^16^ Conversely, NRF2 was also observed to suppress lipogenic gene expression in livers of mice fed a high-fat diet,^17^ underscoring that its function in the regulation of lipid metabolism is complex and context-dependent.

Here, we report a role for NRF2 in the regulation of fatty acid storage in LDs. We find that exposure of cells to exogenous fatty acids leads to the activation of NRF2 and NRF2 activation, which leads to subsequent induction of the protein G0S2, an inhibitor of the lipase ATGL.^18^ Stabilization of NRF2, pharmacological ATGL inhibition, and G0S2-overexpression are sufficient to increase LD content and protect cells against lipotoxic stress and the induction of ferroptosis. We propose that NRF2-induced suppression of TAG hydrolysis is a mechanism to sequester potentially damaging fatty acids to protect cells against lipotoxicity and ferroptosis.

## Results

### NRF2 stability and activity influences LD accumulation in HEK-TtH cells

As NRF2 has previously been observed to alter lipogenic gene expression,^14^^,^^15^^,^^16^^,^^17^ we sought to determine if alteration in the expression of NRF2 plays a role in the regulation of LDs. To test this, we measured LD abundance in HEK-TtH immortalized human embryonic kidney cells^19^ following stabilization or depletion of NRF2.

**Figure 1:**
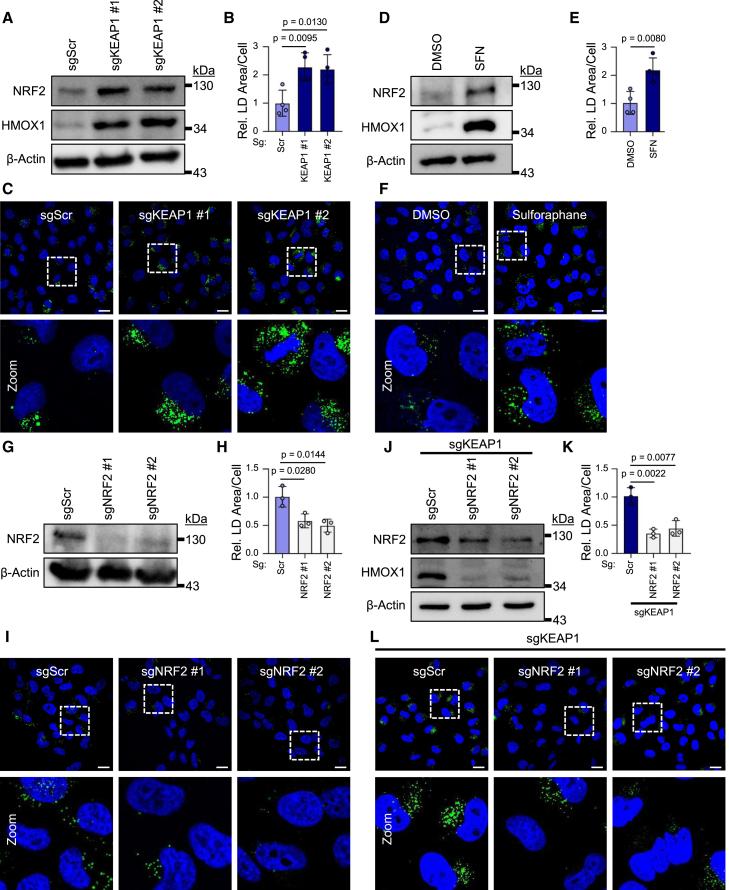
NRF2 stability and activity influences LD accumulation in HEK-TtH cells (A) Immunoblot confirming NRF2 activation in sgKEAP1 cells. (B) Quantification of LD area per cell from (C). *n* = 4 biological replicates. Mean ± SD. (C) Representative images of LDs in sgKEAP1 HEK-TtH cells taken with 63× objective. Scale bars represent 20 μm. (D) Immunoblot confirming NRF2 activation in cells stimulated with 10 μM Sulforaphane for 24 h. (E) Quantification of LD area per cell from (F). *n* = 4 biological replicates. Mean ± SD. (F) Representative images of LDs in HEK-TtH cells treated with 10 μM sulforaphane for 24 h taken with 63× objective. Scale bars represent 20 μm. (G) Immunoblot confirming NRF2 depletion in sgNRF2 cells. (H) Quantification of LD area per cell from (I). *n* = 3 biological replicates. Mean ± SD. (I) Representative images of LDs in sgNRF2 HEK-TtH cells taken with 63× objective. Scale bars represent 20 μm. (J) Immunoblot confirming NRF2 depletion and inactivation of transcriptional activity in sgKEAP1 cells with addition of sgNRF2. (K) Quantification of LD area per cell from (L). *n* = 3 biological replicates. Mean ± SD. (L) Representative images of LDs in sgKEAP1/sgNRF2 double-KO HEK-TtH cells taken with 63× objective. Scale bars represent 20 μm.

In conditions of low stress, NRF2 is bound by the adapter protein Kelch-like ECH-associated protein 1 (KEAP1)^20^ and the E3 ubiquitin ligase Cullin 3 (CUL3),^21^ resulting in ubiquitination and proteasomal degradation. In conditions of oxidative or electrophilic stress, modification of cysteine residues on KEAP1 prevents effective NRF2 ubiquitination, leading to its stabilization, nuclear translocation and transcriptional activity.^22^^,^^23^ To genetically stabilize NRF2, we stably infected HEK-TtH cells with Cas9 and sgRNAs targeting two independent KEAP1 target sequences or a scrambled control sequence. Immunoblot analysis confirms robust stabilization of NRF2 in sgKEAP1 cell lines, as well as increased expression of its transcriptional target, HMOX1 (Figure 1A). We further confirmed mutagenesis of KEAP1 through genomic sequencing at these sgKEAP1 target sites (Figures S1A and S1B). Intriguingly, confocal analysis of LD content through BODIPY 493/503 staining indicates that KEAP1 mutated cells exhibit increased LD abundance (Figures 1B and 1C), providing evidence that NRF2 activity positively regulates LD levels. Consistent with this, treatment of HEK-TtH cells with the drug sulforaphane (SFN),^24^^,^^25^ which stabilizes and activates NRF2 (Figures 1D, S1C, and S1D), and also leads to increased LD levels (Figures 1E and 1F).

To determine the impact of depleting NRF2 levels on LD abundance, we generated pooled CRISPR-Cas9 HEK-TtH cells targeting two independent exons of the *NFE2L2* gene, referred to hereon as sgNRF2, or a scrambled control sequence. sgNRF2 cells exhibit decreased basal NRF2 expression compared to the sgScramble (sgScr) control (Figure 1G) as well as decreased SFN-induced NRF2 and HMOX1 expression (Figure S1E). sgNRF2 cells also exhibit a significant decrease in LD content (Figures 1H and 1I), further supporting a role for NRF2 in the regulation of LD content.

To further validate that the loss of KEAP1 regulates LDs through the stabilization of NRF2, we mutated the *NFE2L2* gene in sgKEAP1 HEK-TtH cells, which exhibit reduced NRF2 and HMOX1 expression (Figure 1J). Loss of NRF2 in sgKEAP1 cells rescues the increase in LD content observed in sgKEAP1 cells (Figures 1K and 1L), indicating that NRF2 is required for the phenotype observed in sgKEAP1 cells and further supporting its role in regulating LD abundance.

### NRF2-mediated LD accumulation is observed in lung cancer cells

**Figure 2:**
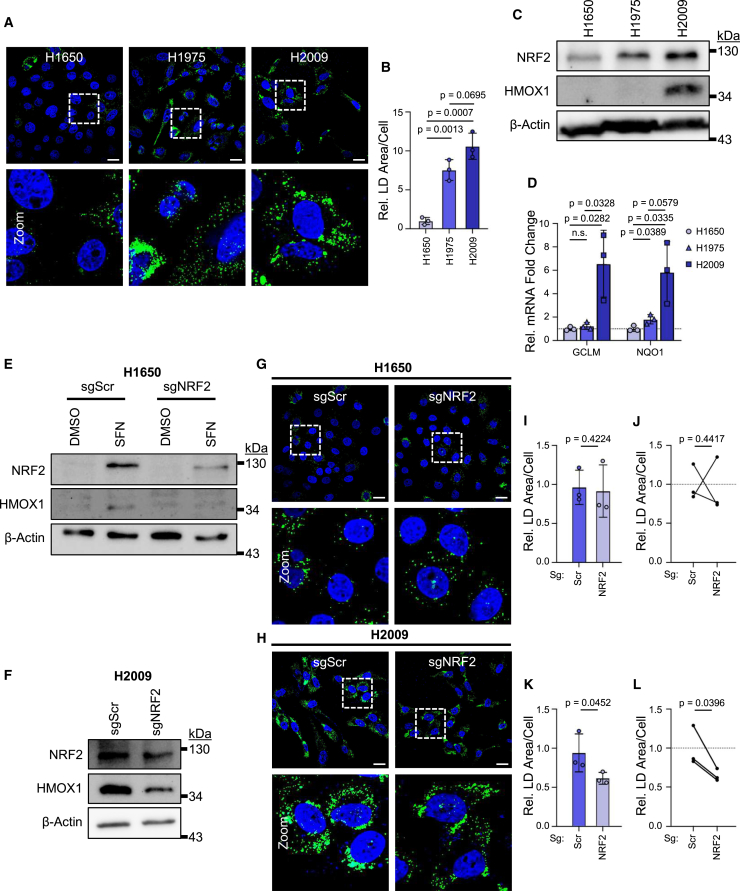
NRF2-mediated LD accumulation occurs in specific lung cancer cell lines (A) Representative images of LDs in lung cancer cells taken with 63× objective. Scale bars represent 20 μm. (B) Quantification of LD area per cell from (A). *n* = 3 biological replicates. Mean ± SD. (C) Immunoblot of basal NRF2 and HMOX1 levels in lung cancer cell lines. (D) Relative basal mRNA expression (2-ΔΔCT relative to GAPDH) of NRF2 transcriptional targets in lung cancer cell lines. Data normalized to H1650 mRNA expression. Horizontal dotted line represents no change relative to H1650 mRNA levels. *n* ≥ 3 biological replicates. Mean ± SD. (E) Immunoblot confirming NRF2 depletion in sgNRF2 H1650 cells. (F) Immunoblot confirming NRF2 depletion in sgNRF2 H2009 cells. (G) Representative images of LDs in sgNRF2 H1650 cells taken with 63× objective. Scale bars represent 20 μm. (H) Representative images of LDs in sgNRF2 H2009 cells taken with 63× objective. Scale bars represent 20 μm. (I) Quantification of LD area per cell from (G). *n* = 3 biological replicates. Statistics represented calculated by Student’s *t* test (one-tailed, unpaired with equal variance). Mean ± SD. (J) Quantification of LD area per cell from (G). Horizontal dotted line represents no change relative to sgScr cells. *n* = 3 biological replicates. Statistics represented calculated by Student’s *t* test (one-tailed, and paired). Mean ± SD. (K) Quantification of LD area per cell from (H). *n* = 3 biological replicates. Statistics represented calculated by Student’s *t* test (one-tailed, unpaired with equal variance). Mean ± SD. (L) Quantification of LD area per cell from (H). Horizontal dotted line represents no change relative to sgScr cells. *n* = 3 biological replicates. Statistics represented calculated by Student’s *t* test (one-tailed, and paired). Mean ± SD.

To determine whether NRF2-mediated LD accumulation is observed in additional contexts, we examined human lung cancer cells, as lung tumors often exhibit mutations or epigenetic alterations that lead to gain of NRF2 function.^26^ Analysis of LD content in the lung cancer cell lines H1650, H1975, and H2009 revealed that H1650 cells have relatively low LD content, while H1975 and H2009 cells had a 7.5-fold and 10.6-fold increase in LD content respectively (Figures 2A and 2B). These data are consistent with previous work demonstrating low LD levels, measured through Oil Red O staining, in H1650 cells and higher levels in H1975 cells.^27^ H1650 cells exhibit low expression NRF2 and its target HMOX1, while H2009 cells exhibit high levels of both proteins. H1975 express moderate levels of NRF2, but no detectable downstream HMOX1 expression (Figure 2C). Consistent with the immunoblot data, H2009 cells, but not H1650 or H1975, exhibit high mRNA levels of NRF2 transcriptional targets glutamate-cysteine ligase modifier subunit (GCLM) and NAD(P)H quinone dehydrogenase 1 (NQO1)^28^ (Figure 2D).

To determine whether the differential expression and activity of NRF2 contribute to the difference in LD content between H1650 and H2009 cells, we used CRISPR to target the *NFE2L2* gene (Figures 2E and 2F) then visualized LDs (Figures 2G and 2H). Deletion of NRF2 had no significant impact on LD content in H1650 cells (Figures 2I and 2J), but led to a moderate but statistically robust decrease in LD content in H2009 cells (Figures 2K and 2L), consistent with the model that increased basal NRF2 activity in H2009 cells contributes to increased LD content.

### The negative regulator of adipose triglyceride lipase, G0S2, acts downstream of NRF2 to regulate LD abundance in HEK-TtH cells

To identify genes that may be involved in this NRF2-mediated LD content accumulation we measured the gene expression proteins of LD-associated perilipins (PLINs), fatty acid regulators, as well as those involved in regulating triacylglycerol esterification or degradation in sgKEAP1 cells.

**Figure 3:**
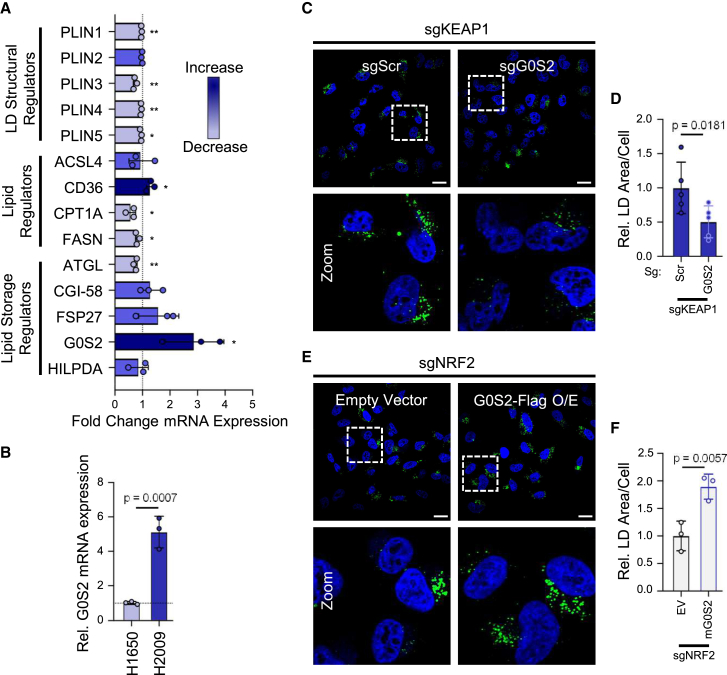
The negative regulator of adipose triglyceride lipase, G0S2, acts downstream of NRF2 to regulate LD abundance in HEK-TtH cells (A) Fold change of mRNA expression (2-ΔΔCT relative to GAPDH) of regulators of LD structure, lipids, and lipid storage in sgKEAP1 cells vs. sgScr cells. Vertical dotted line represents no change relative to sgScr control mRNA levels. *n* = 3 biological replicates. Mean ± SD. (*p* < 0.05 = ∗, *p* < 0.01 = ∗∗). (B) Fold change of mRNA expression (2-ΔΔCT relative to GAPDH) of G0S2 in lung cancer cell lines. Data represented normalized to G0S2 expression of H1650 cells. Horizontal dotted line represents no change relative to H1650 mRNA levels. *n* = 3 biological replicates. Mean ± SD. (C) Representative images of LDs in sgKEAP1/sgG0S2 double-KO HEK-TtH cells taken with 63× objective. Scale bars represent 20 μm. (D) Quantification of LD area per cell from (C). *n* = 5 biological replicates. Statistics represented calculated by Student’s *t* test (one-tailed, unpaired with equal variance). Mean ± SD. (E) Representative images of LDs in sgNRF2/mG0S2-Flag-overexpression HEK-TtH cells taken with 63× objective. Scale bars represent 20 μm. (F) Quantification of LD area per cell from (E). *n* = 3 biological replicates. Statistics represented calculated by Student’s *t* test (one-tailed, unpaired with equal variance). Mean ± SD.

Our analysis of perilipin expression in sgKEAP1 cells revealed small but significant decreases in PLIN1, PLIN4, and PLIN5 compared to control cells, with a larger decrease (27%) in PLIN3, which has been shown to coat the surface of nascent LDs^29^ (Figure 3A). As perilipin proteins are involved in the biogenesis and stability of LDs, their decreased expression is unlikely to contribute to the increased LD burden we observe following NRF2 activation. We also observed a small decrease in the expression of carnitine palmitoyltransferase 1A (CPT1A) and fatty acid synthase (FASN), genes involved in β-oxidation^30^ and fatty acid synthesis,^31^ respectively. following knockout of KEAP1. Additionally, sgKEAP1 cells exhibit a slight increase in expression of the fatty acid translocase, cluster of differentiation 36 (CD36),^16^ consistent with a previous study showing that CD36 supports NRF2-induced LD accumulation through increased fatty acid import (Figure 3A). We note, however, that overall expression of CD36 in HEK-TtH cells is low, even following the increase, and is thus unlikely to be a major contributor to increased LD content.

Next, we assessed the expression of additional genes involved in the breakdown of triacylglycerol from LDs. There are four previously described regulators of ATGL (PNPLA2). CGI-58 (ABHD5)^32^^,^^33^ activates ATGL, while G0S2 and HILPDA (HIG2) inhibit its hydrolase activity.^5^^,^^18^ In conjunction with inhibiting ATGL, HILPDA also stimulates the storage of TAG through the stimulation of DGAT1 activity.^6^ Additionally, FSP27 (CIDEC) has been observed to influence lipolysis activity as well as facilitate the fusion of LDs.^34^^,^^35^^,^^36^^,^^37^^,^^38^ Notably, we observe a significant increase in G0S2 and a depletion of ATGL expression in sgKEAP1 cells compared to sgScramble, but no detectable change in expression of CGI-58, HILPDA, or FSP27 (Figure 3A). Collectively, analysis of genes involved in LD structure and function reveals that NRF2 induction potentially impacts LD content in multiple ways. Because the induction of G0S2 was the most robust response we observed, and because its previously described regulation of ATGL activity is consistent with the increased in LD content we observe, we chose to further explore whether this protein may be involved in LD regulation downstream of NRF2. Consistent with this hypothesis, we observe a 5.1-fold increase in H2009 cells relative to H1650 cells (Figure 3B), suggesting that G0S2 may be one of the links between NRF2 activity and LD phenotype observed in these cells (Figures 2A and 2B).

To determine whether G0S2 induction contributes to LD accumulation in sgKEAP1 cells, we generated a pooled sgG0S2 mutant cell line using CRISPR-Cas9 (Figures S2A and S2B). We observe a decrease in LD levels following G0S2 depletion in sgKEAP1 cells (Figures 3C and 3D), consistent with it playing a functional role downstream of NRF2. Further, stable expression of C-terminal Flag-tagged mouse G0S2 (mG0S2) in sgNRF2 cells (Figures S2C–S2E) significantly increases LD content (Figures 3E and 3F), demonstrating that G0S2 overexpression is sufficient to increase LDs in cells that lack NRF2. We note that loss of G0S2 alone does not reduce LD content in the absence of NRF2 depletion (Figures S2F–S2H), suggesting its role in increasing LD content is limited to conditions in which NRF2 is stabilized. Furthermore, viral over-expression of mG0S2 in WT HEK-TtH cells is not sufficient to increase LD content (Figures S3A–S3C), while transient transfection of mG0S2 is sufficient to increase LD content relative to empty vector control (Figures S3D–S3F), suggesting that G0S2, when expressed at high enough levels, is sufficient to induce LD content in the absence of other NRF2-dependent regulation.

### Specific fatty acids stabilize NRF2 which drives downstream signaling and protects cells against lipotoxic stress

**Figure 4:**
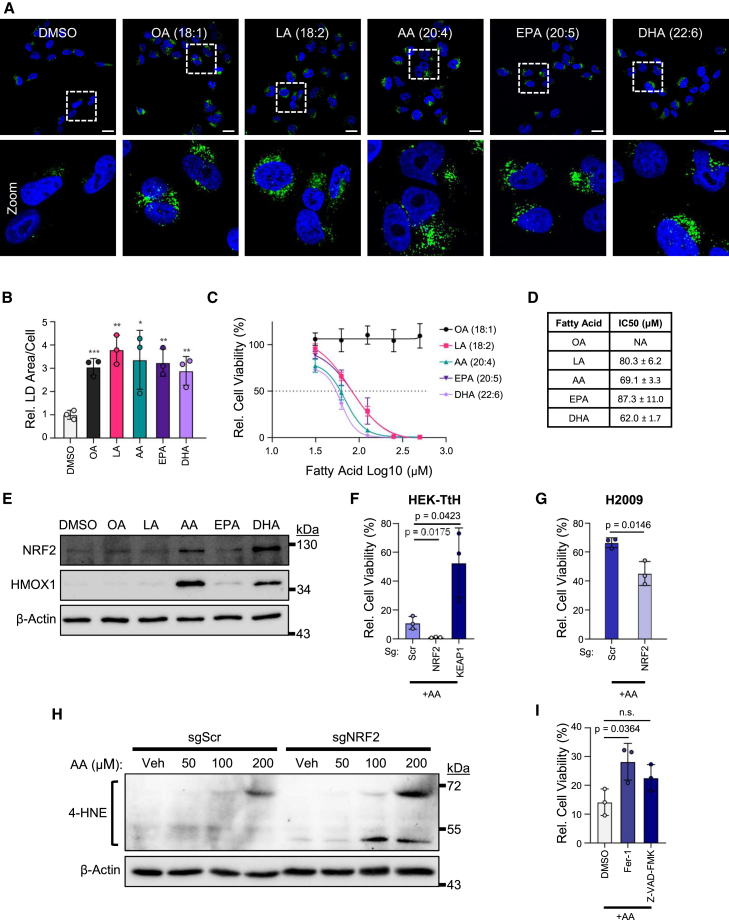
Specific fatty acids stabilize NRF2 and drive downstream signaling to protect cells from lipotoxic stress (A) Representative images of LDs in HEK-TtH cells treated with fatty acids conjugated to fatty acid-free BSA for 4 h taken with 63× objective. Scale bars represent 20 μm. (B) Quantification of LD area per cell from (A). *n* = 3 independent biological replicates. Mean ± SD. (*p* < 0.05 = ∗, *p* < 0.01 = ∗∗, *p* < 0.001 = ∗∗∗). (C) Quantification of cell viability based on luminescence from CellTiter-Glo Luminescent Cell Viability Assay for HEK-TtH cell treated with gradient of fatty acids conjugated to fatty acid-free BSA relative to vehicle controls for 24 h. Horizontal dotted line represents half maximal inhibitory concentration (IC50). *n* = 3 biological replicates of each. (D) IC50 and standard error calculated from (C) for each fatty acid treatment. (E) Immunoblot of NRF2 and downstream transcriptional target HMOX1 after 4 h of treatment of fatty acids conjugated to fatty acid-free BSA. (F) Quantification of cell viability based on luminescence from CellTiter-Glo Luminescent Cell Viability Assay for HEK-TtH sgNRF2 and sgKEAP1 cell lines treated with 200 μM arachidonic acid (AA) conjugated to fatty acid-free BSA for 24 h. *n* = 3 biological replicates of each. Mean ± SD. (G) Quantification of cell viability based on luminescence from CellTiter-Glo Luminescent Cell Viability Assay for H2009 sgNRF2 lung cancer cell lines treated with 200 μM arachidonic acid (AA) conjugated to fatty acid-free BSA for 24 h. *n* = 3 biological replicates of each. Mean ± SD. (H) Immunoblot comparing 4-hydroxynonenal in sgScr and sgNRF2 cells after 4 h treatment with gradient of arachidonic acid conjugated to fatty acid-free BSA. (I) Quantification of cell viability based on luminescence from CellTiter-Glo Luminescent Cell Viability Assay of WT HEK-TtH cells treated with 200 μM arachidonic acid (AA) conjugated to fatty acid-free BSA, co-treated with 10 μM Ferrostatin-1 (Fer-1) or 10 μM Z-VAK-FMK for 24 h. *n* = 3 biological replicates of each. Mean ± SD.

Previous studies have demonstrated that exogenous fatty acids stabilize NRF2 in a number of different cell types.^39^^,^^40^^,^^41^ Based on our observation that NRF2 activity influences LD abundance, we hypothesized that HEK-TtH cells would respond to fatty acid exposure by activating NRF2 as a mechanism to protect cells against lipotoxicity. Initially we evaluated the ability of cells to increase LD content after fatty acids exposure. Cells were grown in media supplemented with fatty acids conjugated to fatty acid–free BSA and imaged prior to the onset of cell death. Confocal analysis indicates that the supplementation of all fatty acid species tested with varying levels of saturation increases LD abundance (Figures 4A and 4B), validating that HEK-TtH cells will readily incorporate these particular free fatty acids into LDs. Intriguingly, the monounsaturated fatty acid (MUFA) oleic acid (OA, 18:1) is well tolerated, but the polyunsaturated fatty acids (PUFAs) arachidonic acid (AA, 20:4) and docosahexaenoic acid (DHA, 22:6) significantly decrease cell viability when fed to cells over a range of doses (Figures 4C, 4D, and S4A). This decrease in cell viability correlates with stabilization of NRF2 and expression of its target HMOX1 (Figure 4E). Furthermore, linoleic acid (LA, 18:2) and eicosapentaenoic acid (EPA, 20:5) are better tolerated, and do not induce NRF2 stabilization at the concentrations tested (Figure S4B). Altogether, these data provide evidence that select PUFAs stimulate NRF2 stability and activity.

Next, to determine the contribution of NRF2 to cytoprotection upon exposure to fatty acids we measured viability of sgNRF2 and sgKEAP1 HEK-TtH cells and sgNRF2 H2009 cells treated with AA (Figures 4F and 4G, respectively). Notably, the viability of sgNRF2 cells treated with AA is reduced, whereas sgKEAP1 cell viability is increased relative to the sgScr controls. Furthermore, we observed similar NRF2-mediated protection in DHA treated HEK-TtH sgNRF2 and sgKEAP1 cell lines (Figure S4C). Consistent with a role for NRF2 in protection against AA-induced lipotoxicity, sgNRF2 HEK-TtH cells exhibit increased staining for 4-hydroxynonenal (4-HNE), a product of lipid peroxidation, compared to sgScr cells following AA treatment (Figure 4H). Additionally, we find that AA-induced toxicity is decreased in AA-treated cells co-treated with the ferroptosis inhibitor Ferrostatin-1^42^ (Fer-1) but not the apoptosis inhibitor, Z-VAD-FMK (Figure 4I). This is in contrast to DHA treated HEK-TtH cells, which are dying through an apoptotic mechanism (Figure S4D). Collectively, these data are consistent with a model in which cellular exposure to the PUFA arachidonic acid leads to stabilization of NRF2, which in turn reduces lipid peroxidation and subsequent ferroptosis.

### Inhibition of lipid droplet degradation protects cells from ferroptosis

**Figure 5:**
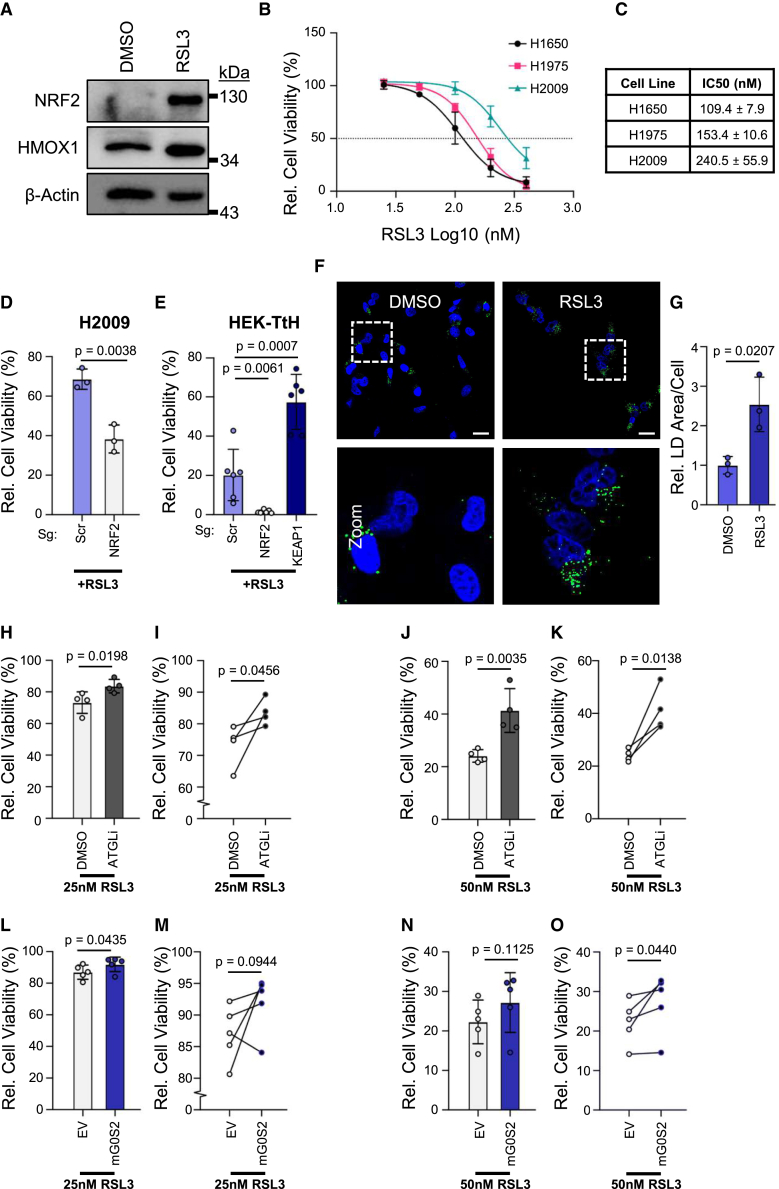
Inhibition of lipid droplet degradation protects cells from ferroptosis (A) Immunoblot of NRF2 stability and downstream transcriptional target HMOX1 after treatment with 200 nM RSL3 for 4 h. (B) Quantification of cell viability based on luminescence from CellTiter-Glo Luminescent Cell Viability Assay for HEK-TtH cell treated with gradient of RSL3 relative to DMSO controls for 24 h. Horizontal dotted line represents half maximal inhibitory concentration (IC50). *n* = 4 biological replicates of each. (C) IC50 and standard error calculated from (B) for each lung cancer cell line treated with RSL3 gradient. (D) Quantification of luminescence from CellTiter-Glo Luminescent Cell Viability Assay of sgNRF2 H2009 cell lines treated with 200 nM RSL3 relative to vehicle control for 24 h. *n* = 3 biological replicates of each. Mean ± SD. (E) Quantification of luminescence from CellTiter-Glo Luminescent Cell Viability Assay of sgNRF2 and sgKEAP1 HEK-TtH cell lines treated with 200 nM RSL3 relative to vehicle control for 24 h. *n* = 6 biological replicates of each. Mean ± SD. (F) Representative images of LDs of HEK-TtH cells treated with 50 nM RSL3 for 24 h taken with 63× objective. Scale bars represent 20 μm. (G) Quantification of LD area per cell from (F). *n* = 3 biological replicates. Mean ± SD. (H) Quantification of cell viability from CellTiter-Glo Glo Luminescent Cell Viability Assay of sgNRF2 HEK-TtH cells treated with DMSO or 10 μM ATGListatin (ATGLi) in combination with 25 nM RSL3. *n* = 4 biological replicates. Statistics represented calculated by Student’s *t* test (one-tailed, unpaired with equal variance). Mean ± SD. (I) Representation of data from (H) when paired within each individual biological replicate. Statistics represented calculated by Student’s *t* test (one-tailed, paired). Mean ± SD. (J) Quantification of cell viability from CellTiter-Glo Glo Luminescent Cell Viability Assay of sgNRF2 HEK-TtH cells treated with DMSO or 10 μM ATGListatin (ATGLi) in combination with 50 nM RSL3. *n* = 4 biological replicates. Statistics represented calculated by Student’s *t* test (one-tailed, unpaired with equal variance). Mean ± SD. (K) Representation of data from (J) when paired within each individual biological replicate. Statistics represented calculated by Student’s *t* test (one-tailed, paired). Mean ± SD. (L) Quantification of cell viability from CellTiter-Glo Glo Luminescent Cell Viability Assay of sgNRF2 HEK-TtH cells overexpressing mG0S2-Flag or empty vector (EV) treated with 25 nM RSL3. *n* = 5 biological replicates. Statistics represented calculated by Student’s *t* test (one-tailed, unpaired with equal variance). Mean ± SD. (M) Representation of data from (L) when paired within each individual biological replicate. Statistics represented calculated by Student’s *t* test (one-tailed, paired). Mean ± SD. (N) Quantification of cell viability from CellTiter-Glo Glo Luminescent Cell Viability Assay of sgNRF2 HEK-TtH cells overexpressing mG0S2-Flag or empty vector (EV) treated with 50 nM RSL3. *n* = 5 biological replicates. Statistics represented calculated by Student’s *t* test (one-tailed, unpaired with equal variance). Mean ± SD. (O) Representation of data from (N) when paired within each individual biological replicate. Statistics represented calculated by Student’s *t* test (one-tailed, paired). Mean ± SD.

To further explore the relationship between LDs, NRF2, and ferroptosis, we treated HEK-TtH cells with RSL3,^43^ an inhibitor of glutathione peroxidase 4 (GPX4),^44^ a key enzyme involved in reducing lipid peroxide radicals.^45^ Without a functional GPX4 enzyme, lipid peroxide radicals accumulate and induce ferroptosis.^44^ Consistent with this, cell death upon RSL3 treatment is fully abolished in HEK-TtH cells by simultaneous treatment with the lipid radical scavenger, Ferrostatin-1,^46^ but not the caspase inhibitor Z-VAD-FMK (Figure S5A). Importantly, RSL3 treatment stabilizes NRF2 and induces HMOX1 expression prior to the induction of cell death (Figure 5A). Additionally, we evaluated the effectiveness of the RSL3 on lung cancer cell lines (Figures 5B and 5C) and observe that H2009 cells, which have a high NRF2-signature and high basal LD levels are more resistant to RSL3 than H1650 cells, which have a low NRF2-signature and low basal LD levels. H1975 cells which have a relatively low NRF2-signature but high LD levels, have increased resistance to RSL3-induced death relative to H1650, further supporting that increased LD storage is protective against ferroptosis. Furthermore, sgNRF2 H2009 cells exhibit an increased sensitivity to RSL3-induced death (Figure 5D), while sgNRF2 and sgKEAP1 HEK-TtH cells exhibit increased and decreased sensitivity to RSL3, respectively (Figure 5E), altogether supporting the critical role NRF2 has in protecting cells from ferroptotic cell death.

Our data suggest that cells increase LD storage as a protective mechanism against ferroptosis. To further test this, we treated HEK-TtH cells with a low dose of RSL3 and performed confocal microscopy (Figure 5F). Intriguingly, although there is substantial cell death following treatment, cells that survive exhibit significantly increased LD content relative to DMSO control treated cells (Figure 5G). Additionally, we developed a population of RSL3-resistant HEK-TtH cells by treating with an increasing dose of RSL3, similar to a previous study using head and neck cancer cells,^47^ and confirmed resistance over a range of RSL3 doses (Figures S5B and S5C). RSL3-resistant cells exhibit increased NRF2 stability and downstream activity (Figure S5D), as well as a significant increase in LD content relative to cells continuously maintained in media supplemented only with DMSO (Figures S5E and S5F). Furthermore, these cells maintain resistance (Figures S5G and S5H) and increased LD storage upon RSL3 removal (Figures S5I and S5J). These findings indicate that sequestration of fatty acids in LDs is a potential adaptation to overcome chronic lipotoxic stress and ferroptosis.

Our model suggests that cells decrease lipolysis activity as a mechanism to protect against ferroptosis. To test whether inhibition of lipolysis is sufficient to protect against ferroptosis, we treated cells with the drug ATGListatin (ATGLi),^48^ a selective inhibitor of ATGL, to suppress the breakdown of TAG and mimic the function of NRF2-induced LD storage. Importantly, ATGLi treatment increases LD content (Figures S5K and S5L), confirming the drug is active against ATGL and confirming ATGL inhibition is sufficient to induce LD accumulation. We then treated cells with a combination of ATGLi and RSL3 and observed that ATGLi treatment leads to increased viability of RSL3 treated sgNRF2 cells (Figures 5H–5K). Additionally, we measured the viability of RSL3 treated sgNRF2 HEK-TtH cells engineered to overexpress mG0S2 and observed a similar rescue in viability (Figures 5L–5O). These data support our hypothesis that blocking the release of fatty acids stored as TAG from LDs is a mechanism through which cells prevent the release of potentially toxic fatty acids.

Collectively, our data provide evidence that NRF2 is stabilized by lipotoxic stress and promotes changes in gene expression that favor increased LD storage. Among the genes induced is the ATGL suppressor G0S2, whose activity limits TAG breakdown to prevent the release of potentially toxic fatty acids. We propose that this function of NRF2 is one of multiple stress response mechanisms the cell uses to limit cellular toxicity and maintain cell viability.

## Discussion

NRF2 responds to diverse oxidative and electrophilic stresses and mounts a broad cytoprotective transcriptional response. In this work, we have identified that NRF2 responds to fatty acid exposure and lipotoxic stress by preventing the breakdown of LDs to sequester potentially toxic fatty acids. Mechanistically, NRF2 promotes this increased fatty acid storage, at least in part, by promoting increased expression of the protein G0S2, an inhibitor of the rate-limiting mediator of lipolysis, ATGL. Though current evidence does not support that NRF2 directly induces G0S2 transcription, we find that the increased sensitivity of NRF2 depleted cells to the induction of ferroptosis is partially rescued by pharmacological inhibition of ATGL, indicating that this NRF2-dependent activity is an important protective mechanism. Collectively, our data support a model in which NRF2 senses lipotoxic stress and responds by suppressing TAG hydrolysis to limit cellular exposure to fatty acids.

The ability to sense and respond to lipotoxic stress is critical to maintain cellular homeostasis, and disruption of the lipotoxic stress response is associated with diverse pathological conditions. The role of NRF2 as a key mediator of the lipotoxic stress response is well established, as exposure to exogenous fatty acids^39^^,^^40^^,^^41^ and pharmacological induction of ferroptosis^47^ lead to NRF2 stabilization and subsequent transcriptional activation of genes whose function is to limit the accumulation of lipid peroxides. Our finding that NRF2 promotes the inhibition of TAG hydrolysis and prevents the release of free fatty acids from LD stores is consistent with this function and adds an additional mechanism through which its cytoprotective effects are mediated.

The identification of a relationship between NRF2 and LD dynamics has potential consequences for our understanding of health and disease. The ability of NRF2 to promote lipid storage may influence the ability of cells to utilize lipids for cellular processes such as membrane biogenesis or fatty acid oxidation. Notably, activation of NRF2 occurs in several malignancies through a variety of mechanisms, including mutations in KEAP1 or NRF2 that disrupt their interaction, epigenetic changes that influence NRF2 and KEAP1 expression, and chronic stress within the tumor microenvironment.^49^ Increased NRF2 expression and activity have been shown to play roles in cancer initiation and progression,^50^^,^^51^ metastasis,^52^ chemoresistance,^53^ and metabolism.^54^ Importantly, LD dynamics have also been shown to be altered in diverse tumor types^55^ and can be either tumor suppressive,^56^ or promote tumor growth and survival^57^ and chemoresistance,^58^^,^^59^ depending on context. We speculate that the regulation of LD dynamics by NRF2 may represent one of its functions associated with the promotion of tumor growth and this relationship may present new opportunities for therapeutic intervention.

### Limitations of the study

At present, we cannot rule out additional mechanisms through which NRF2 activity promotes increased LD content or determine how broadly this regulation extends across different cell types and conditions. For example, NRF2 regulates the expression of the fatty acid transporter CD36 in pancreatic β-cells.^16^ NRF2 also induces expression of the autophagy regulator p62.^60^ As stress-induced autophagy increases LD content through the breakdown of membrane phospholipids,^61^ it is possible that the crosstalk between NRF2 and autophagy is contributing to the phenotypes we observe. Future studies are needed to fully understand the full scope of potential mechanisms through which NRF2 regulates LD dynamics under lipotoxic and other stress conditions.

Importantly, though our study identifies that NRF2 stabilization promotes increased transcription of G0S2, our data do not support that G0S2 is a direct transcriptional target of NRF2. However, NRF2 has been shown to directly activate transcription of peroxisome proliferator-activated receptor gamma (PPARγ),^62^ and PPARγ has been shown to directly regulate expression of G0S2.^63^ Thus, we hypothesize that NRF2-mediated G0S2 induction is mediated indirectly through PPARγ, though future work is needed to confirm this link.

Collectively, our study supports a model in which cells respond to lipotoxic stress by increasing expression and stability of NRF2 which inhibits TAG hydrolysis to promote lipid storage and protect cells against specific fatty acids that might otherwise have toxic effects on the cell. This work supports the need for further investigation of the relationship between NRF2 and LD dynamics, and how this relationship impacts health and disease.

## Resource availability

### Lead contact

Further information and requests for resources and reagents should be directed to and will be fulfilled by the lead contact, Dr. David Kashatus (kashatus@virginia.edu).

### Materials availability

Materials generated in this studying including cell lines, and plasmids will be available upon request of the lead contact.

### Data and code availability

• All data reported in this paper will be shared by the lead contact upon request.
• This paper does not report original code.
• Any additional information required to reanalyze the data reported in this paper is available from the lead contact upon request.

## Acknowledgments

We thank Dr. Jun Liu from the Mayo Clinic for providing the Flag tagged mouse G0S2 gene^64^ used in this study and Natalia Dworak and Stacy Criswell from the UVA AMF for help with microscopy. This research was supported by 10.13039/100000054NCI grant 1U54CA274499 (D.F.K.).

## Author contributions

Conceptualization, C.T.P. and D.F.K.; methodology, C.T.P. and D.F.K.; formal analysis, C.T.P. and W.B.G.; investigation, C.T.P. and W.B.G.; resources, D.F.K.; writing – original draft, C.T.P. and D.F.K.; visualization, C.T.P.; supervision, D.F.K.; project administration, C.T.P. and D.F.K.; funding acquisition, D.F.K.

## Declaration of interests

The authors declare no competing interests.

## STAR★Methods

### Key resources table

**Table:** 

REAGENT or RESOURCE	SOURCE	IDENTIFIER
**Antibodies**		

Rabbit monoclonal anti-β-Actin (D6AB)	Cell Signaling Technology	Cat# 8457; RRID: AB_10950489
NRF2 (D1Z9C) XP Rabbit mAB	Cell Signaling Technology	Cat# 12721; RRID: AB_2715528
HMOX1 Antibody, Rabbit Pab	Sino Biological	Cat# 201131-T42
Flag M2 Antiody	Sigma	Cat# F1804; RRID: AB_262044
4-Hydroxynonenal Antibody	Biotechne	Cat# MAB3249; RRID: AB_664165

**Chemicals, peptides, and recombinant proteins**		

BODIPY 493/503	Invitrogen	Cat# D3922
Bis-BENZIMIDE H-33342 TRIHYDROCHLORIDE (Hoechst)	VWR (MP Biomedicals)	Cat# 190305
Trizol Reagent	Thermo Fisher Scientific	Cat# 15596026
iScript cDNA Synthesis Kit	Bio-Rad	Cat# 1708890
Power SYBR Green PCR Master Mix	Fisher Scientific	Cat# 43-687-06
Bovine Serum Albumin (BSA) Fatty Acid-Free Powder	Fisher Scientific	Cat# BP9704-100
Oleic Acid	Caymen Chemical	Cat # 90260
Linoleic Acid	Caymen Chemical	Cat# 90150
Arachidonic Acid	Caymen Chemical	Cat# 90010
Eicosapentaenoic Acid	Caymen Chemical	Cat# 90110
Docosahexaenoic Acid	Caymen Chemical	Cat# 90310
RSL3	MedChemExpress	Cat# HY-100218A
Z-VAD-FMK	MedChemExpress	Cat# HY-16658B
Ferrostatin-1	MedChemExpress	Cat# HY-100579
ATGListatin	Sigma-Aldrich	Cat# SML1075

**Critical commercial assays**		

CellTiter-Glo Luminescent Cell Viability Assay	Promega	Cat# G7571

**Experimental models: Cell lines**		

HEK-TtH	Lim et al.^65^	
NCI-H1650 (H1650)		RRID:CVCL_1483
NCI-H1975 (H1975)		RRID:CVCL_1511
NCI-H2009 (H2009)		RRID:CVCL_1514

**Recombinant DNA**		

mG0S2-Flag	Gifted from Jun Liu Lab; Heckmann et al.^64^	
plentiCRISPRv2	Sanjana et al.^66^	RRID:Addgene 52961
pBabe-Puro	Morgenstern and Land^67^	RRID:Addgene 1764
sgTarget Sequences	Please see Table S1	

**Oligonucleotides**		

Please see Tables S2 and S3		

**Software and algorithms**		

Fiji	ImageJ	https://fiji.sc
GraphPad Prism	GraphPad Inc.	https://www.graphpad.com
Zen Microscopy Software	Zeiss	https://www.zeiss.com/microscopy/us/products/microscope-software/zen.html
Inkscape: Open Source Scalable Vector Graphics Editor	Inkscape	https://inkscape.org/
Ensembl Genome Browser	Ensembl	Ensembl.org
NCBI BLAST tools	NIH	https://blast.ncbi.nlm.nih.gov/Blast.cgi
R	R foundation for Statistical Computing	https://www.R-project.org/
drc (Dose-Response Analysis Using R) package	Ritz et al.^68^	

### Experimental model and study participant details

#### Cell culture

Human Embryonic Kidney cells expressing hTert as well as large and small T-antigen (HEK-TtH, unspecified sex)^19^^,^^65^ were cultured in DMEM media (Gibco #11965-092) and human lung cancer cell lines (H1650 – Male, H1975 – Female, H2009 – Female) were grown in RPMI 1640 media (Gibco #11875-093). Both DMEM and RPMI 1640 media were supplemented with 1x Penicillin/Streptomycin (Gibco #15140-122) and 10% FBS (Gibco #16000-044). Cells were grown in a humified incubator at 37°C with 5% CO_2_. Mycoplasma testing of all cell lines is performed every 6 months through a service provided by the University of Virginia Cell Culture Facility.

### Method details

#### Plasmid generation

##### CRISPR vectors

LentiCRISPRv2 plasmid (Addgene #52961),^66^ or LentiCRISPRv2 plasmid with neomycin resistance cassette in place of Puromycin resistance cassette^69^ were digested with the restriction enzyme BsmBI-v2 (New England Biolabs #R0580) at 55°C. Oligonucleotides containing sgTargets sequences (Table S1) with the addition of overhang nucleotide sequences compatible to destination vector were annealed by slow cooling in T4 DNA Ligase Reaction Buffer (New England Biolabs #B0202S). Annealed oligonucleotides were subsequently inserted into the BsmBI digested and purified LentiCRISPRv2 vectors with T4 DNA ligase (New England Biolabs #M0202T).

##### Overexpression vectors

Overexpression plasmid pBabe-Puro (addgene #1764)^67^ was cut by multiple restriction enzymes, generating incompatible ends. Overexpression genes were amplified by PCR with Phusion High-Fidelity DNA polymerase (New England Biolabs #M0530), adding appropriate restriction enzyme cut sites with at least 6 additional nucleotides to both ends of amplicon. DNA amplicons were purified with QIAquick PCR Purification Kit (QIAGEN #28104), and cut with appropriate restriction enzymes. Restriction digested plasmid and amplicons were run on DNA gel and were isolated by gel extraction using PureLink Quick Gel Extraction Kit (Invitrogen #K210012). Amplicons were then ligated into destination vector with T4 DNA ligase (New England Biolabs #M0202T).

#### Lentivirus and retrovirus generation and transduction

Lentivirus was produced by transfection of HEK-293T cells with the lentiviral plasmids psPAX2 (Addgene #12260), pCMV-VSV-G (Addgene #8454), along with vector containing human immunodeficiency virus type 1 (HIV-1) packaging signal. Retrovirus was produced by transfection of HEK-293T cells with retroviral plasmid pCL-10A1 (Novus Biologicals #NBP2-29542) along with vector containing Moloney murine leukemia virus (MMLV) packaging signal. Media containing virus was isolated by filtration with 0.45 μm syringe filter. Filtered media containing virus was placed on cells for two days along with 1x polybrene (4 μg/ml). Transduced cells were then selected by the addition of 1 μg/ml Puromycin dihydrochloride (Sigma #P8833) or 500 μg/ml Geneticin (Invitrogen #10131-035). Selection occurred alongside uninfected control cells for appropriate amount of time.

#### Genomic sequencing of sgTargets

Genomic DNA was extracted from cells using PureLink Genomic DNA Mini Kit (Invitrogen #K1820-00). Genomic region of interest was amplified by PCR with Phusion High-Fidelity DNA polymerase (New England Biolabs #M0530). PCR amplicons were isolated by gel extraction using PureLink Quick Gel Extraction Kit (Invitrogen #K210012), and were sequenced with custom oligonucleotides (Table S2) by Eurofins Genomics.

#### Cell line transfection

Cells were seeded at 2.5x10^5^ cells in 10 cm tissue culture dishes, or 3x10^4^-4x10^4^ cells/well in each individual 6-well dish with glass coverslips (22 mm x 22 mm) in full media. After 1 overnight, media on cells was replenished with antibiotic free media. Cells were transfected utilizing Lipofectamine 2000 Transfection Reagent (ThermoFisher Scientific #11668027) with 250 ng of plasmid per 1 ml of media.

#### Lipid droplet staining and quantification by microscopy

Glass coverslips (22 x 22 mm) were placed in individual wells of 6-well dish. Coverslips were washed with 1xPBS and dried with vacuum. Cells were then seeded at 3x10^4^-4x10^4^ cells/well in each individual well. After 1 overnight, cells were either treated as indicated, or media was changed. After indicated amount of time, media was removed and cells were fixed by incubating in 3% Paraformaldehyde (Methanol-free) at room temperature for 5 minutes. Lipid droplets within the cells were then stained with a working concentration of 1 μg/ml BODIPY 493/503 (Invitrogen) in 1xPBS for 30 min at room temperature in the dark. Nuclei were then stained with a working concentration of 1 μg/ml Hoechst in 1xPBS for 5 min. Coverslips were then washed twice by incubating at room temperature for 5 min in 1xPBS. Coverslips were mounted onto glass slides with ProLong Gold Antifade Mountant (Thermo Fisher Scientific #P36930).

Cells were imaged at 63x magnification on the Zeiss LSM 710 microscope (Advanced Microscopy Facility, University of Virginia) or the Zeiss LSM 900 microscope (Microbiology, Immunology, and Cancer Biology, University of Virginia). BODIPY z-stack images were taken every 0.5 μm through field of view, and were compressed into orthogonal projection. All imaged were exported as TIFF files for downstream analysis.

Images were analyzed utilizing Fiji software. Total number of cells in a field of view were quantified with “Multi-point” function, counting individual Hoechst-stained nuclei. Total lipid droplet area in individual fields of view were quantified with “Analyze Particles” function. For each individual experiment, the total lipid droplet area from all fields of view imaged was divided by total number of nuclei from all fields of view imaged.

#### Western blot

Cell pellets were suspended in ice-cold RIPA buffer (1% IGEPAL CA-630, 20 mM Tris pH 8, 137 mM NaCl, 10% glycerol, and 2 mM EDTA) containing protease inhibitors (2 μg/ml aprotinin, 2 μg/ml leupeptin, 1 mM PMSF, 1 mM Na_3_VO_4_, and 50 mM NaF) and incubated on ice for 30 min in microcentrifuge tubes. Cells were spun at max speed for 10 min. Cell lysate was then transferred to fresh microcentrifuge tube on ice. Equivalent protein content was determined by Bio-Rad Protein Assay Dye (Bio-Rad #5000006). Protein was resolved by SDS-PAGE, followed by transfer to activated (Methanol soaked) PVDF membrane. Membranes were probed with primary antibody, followed by secondary antibody conjugated to horseradish peroxidase (HRP). HRP signal was activated by the addition of WesternBright chemoluminescent detection reagents (advansta), and detected with ChemiDoc imager (Bio-Rad).

#### RNA extraction

Cells were seeded at 2.5x10^5^ cells in 10 cm tissue culture dishes. After 1 overnight, media on cells was replenished or treated as indicated. After 24 h, cells were harvested and spun down at 300xg for 5 min at 4°C. Supernatant was removed and cell pellets were flash frozen and stored at -80°C until RNA extraction. RNA was extracted from cell pellets utilizing TRIzol Reagent (Thermo Fisher Scientific #15596026), following protocol for RNA extraction provided by the manufacturer.

For transfected cells, following RNA extraction, extract was treated with DNAse I (RNAse-free) (New England Biolabs #M0303) and incubated for 15 min at 37°C. RNA was then purified utilizing RNeasy MinElute Cleanup Kit (QIAGEN #74204).

#### RT-qPCR and RT-PCR

RNA concentration was determined by nano-drop, and 1 μg of RNA was reverse transcribed utilizing iScript cDNA Synthesis kit (Bio-Rad #1708890) in a 20 μl reaction. Quantitative PCR was performed utilizing Power SYBR Green PCR Master Mix (Fisher Scientific #43-687-06). Non-quantitative RT-PCR was performed utilizing MyTaq Red Mix (Bioline #BIO-25043) and subsequent resolving amplicons though gel electrophoresis with 1xTAE agarose gel.

Oligonucleotides sequences used (Table S3) were either previously described,^70^^,^^71^^,^^72^^,^^73^ or designed to specifically bind mRNA, when possible, with oligonucleotides spanning exon-exon junctions. Primer-BLAST of oligonucleotides against Refseq mRNA database of Homo sapiens suggests specificity of target gene transcripts. cDNA specific quantification was validated employing negative control of non-reverse transcribed RNA for each individual sample. Fold change for quantitative PCR was calculated using 2^-ΔΔCt^, utilizing GAPDH sequence^74^ for normalization against gene of interest.

#### Fatty acid supplementation

Fatty acids were conjugated to 1% fatty-acid free Bovine Serum Albumin (FAF-BSA, Fisher Scientific #BP9704-100) in complete media (10% FBS), or media containing 1% FBS. Fatty acid stocks (suspended in ethanol) were diluted to 200 mM in DMSO and solubilized at 70°C for 15 min. Fatty acids were then added to media containing fully dissolved FAF-BSA at 37°C to desired final concentration, mixed by inversion, and incubated for at least 15 min before use. Vehicle controls with appropriate proportion of ethanol and DMSO were generated for individual fatty acid species.

#### CellTiter-Glo luminescent cell viability assay

Seeded 1x10^3^ cells/well in 96-well dish in complete media. After 1 overnight, media was removed and cells were treated in triplicate as indicated. Media was removed and cells were incubated with 50 μl of CellTiter-Glo solution (Promega #G7571) diluted with 1xPBS (2:3) for 10 min in the dark at room temperature. Contents from each well was then transferred to white 96-well dish and luminescence was measured on Perkin Elmer Victor 3 1420 Multilabel Counter. Average luminescence measurement of each individual treatment was normalized to vehicle control.

### Quantification and statistical analysis

GraphPad Prism (version 10.4.1) was used to produce graphical representations of data. Inkscape was used for customization of individual graphs and figure animations. Cell viability data was fit to a 4-parameter logistics model to estimate the half maximal inhibitory concentration (IC_50_) and standard error in R utilizing the drc package^68^ (version 3.0.1). All p-values indicated were calculated with a Student’s *t*-Test. All *t*-Tests calculated were two-tailed and unpaired with equal variance unless otherwise specified. Dispersion and precision measures are indicated on individual graphs with data points and error bars representing standard deviation above and below the mean. When applicable, data represented is normalized to control conditions.
