# Supplementary material for: NRF2 regulates lipid droplet dynamics to prevent lipotoxicity
Source: iScience. 2025 Jun 18;28(7):112925. doi: 10.1016/j.isci.2025.112925 (PMC12270665; doi:10.1016/j.isci.2025.112925)
Supplement: Document S1. Figures S1–S5 and Tables S1–S3 [file mmc1.pdf]

iScience, Volume *28*

Supplemental information

NRF2 regulates lipid droplet dynamics to prevent lipotoxicity

Christopher T. Prevost, William B. Gansereit, and David F. Kashatus

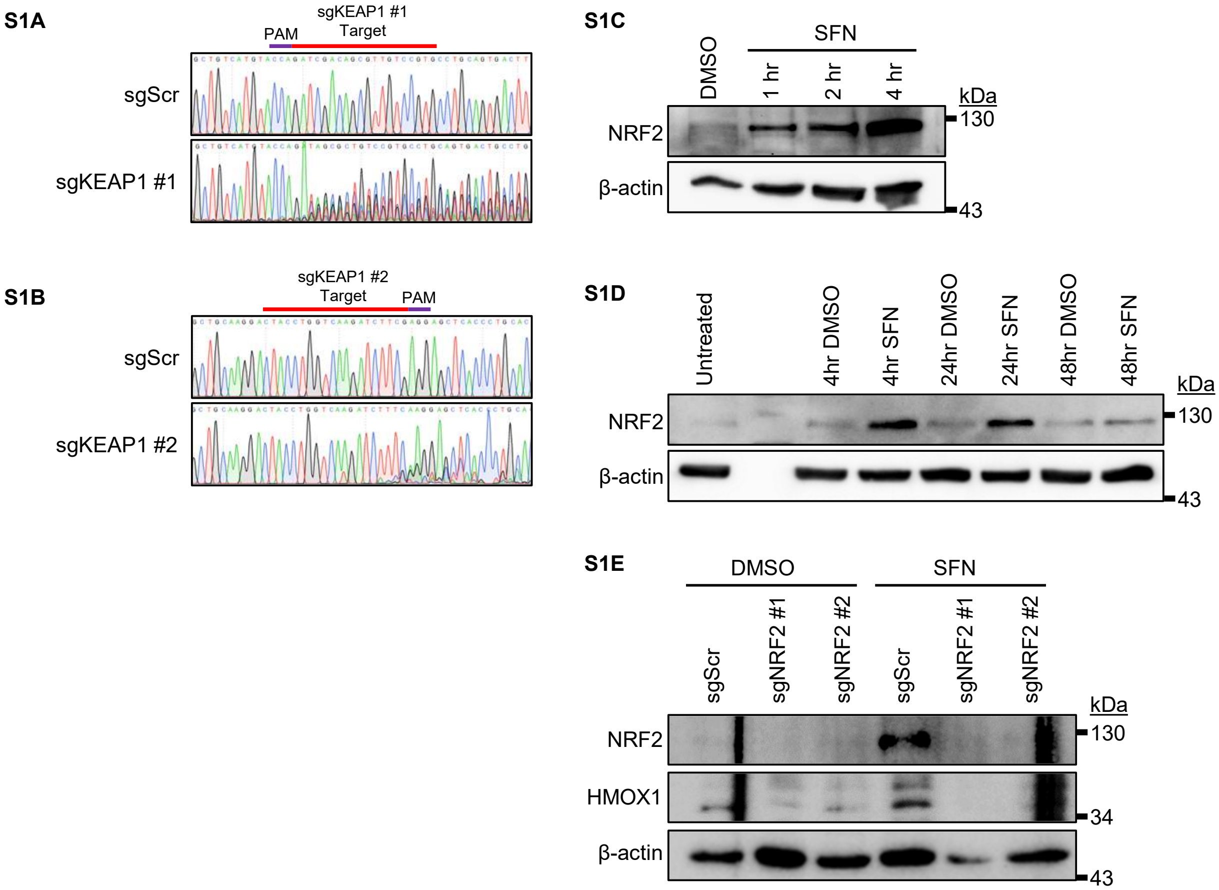

Supplemental Figure 1. Validation of genetic and pharmacological approaches to stabilize NRF2.

(A) Chromatogram of genomic DNA sequencing of the KEAP1 gene in HEK-TtH cells transduced with sgKEAP1 #1 (mixed population) and sgScr (mixed population). sgKEAP1 #1 targets exon 2 at sequence 5’-CACGGACAACGCTGTCGATC-3’.

(B) Chromatogram of genomic DNA sequencing of the KEAP1 gene in HEK-TtH cells transduced with sgKEAP1 #2 (mixed population) and sgScr (mixed population). sgKEAP1 #2 targets exon 3 at sequence 5’-CTACCTGGTCAAGATCTTCG-3’.

(C) Immunoblot for NRF2 in HEK-TtH cells treated with 10μM sulforaphane or DMSO over a 4-hour time course.

(D) Immunoblot for NRF2 in HEK-TtH cells treated with 10μM sulforaphane or DMSO over a 48-hour time course.

(E) Immunoblot for NRF2 and HMOX1 in sgNRF2 and sgScr HEK-TtH cells treated with 10μM sulforaphane or DMSO.

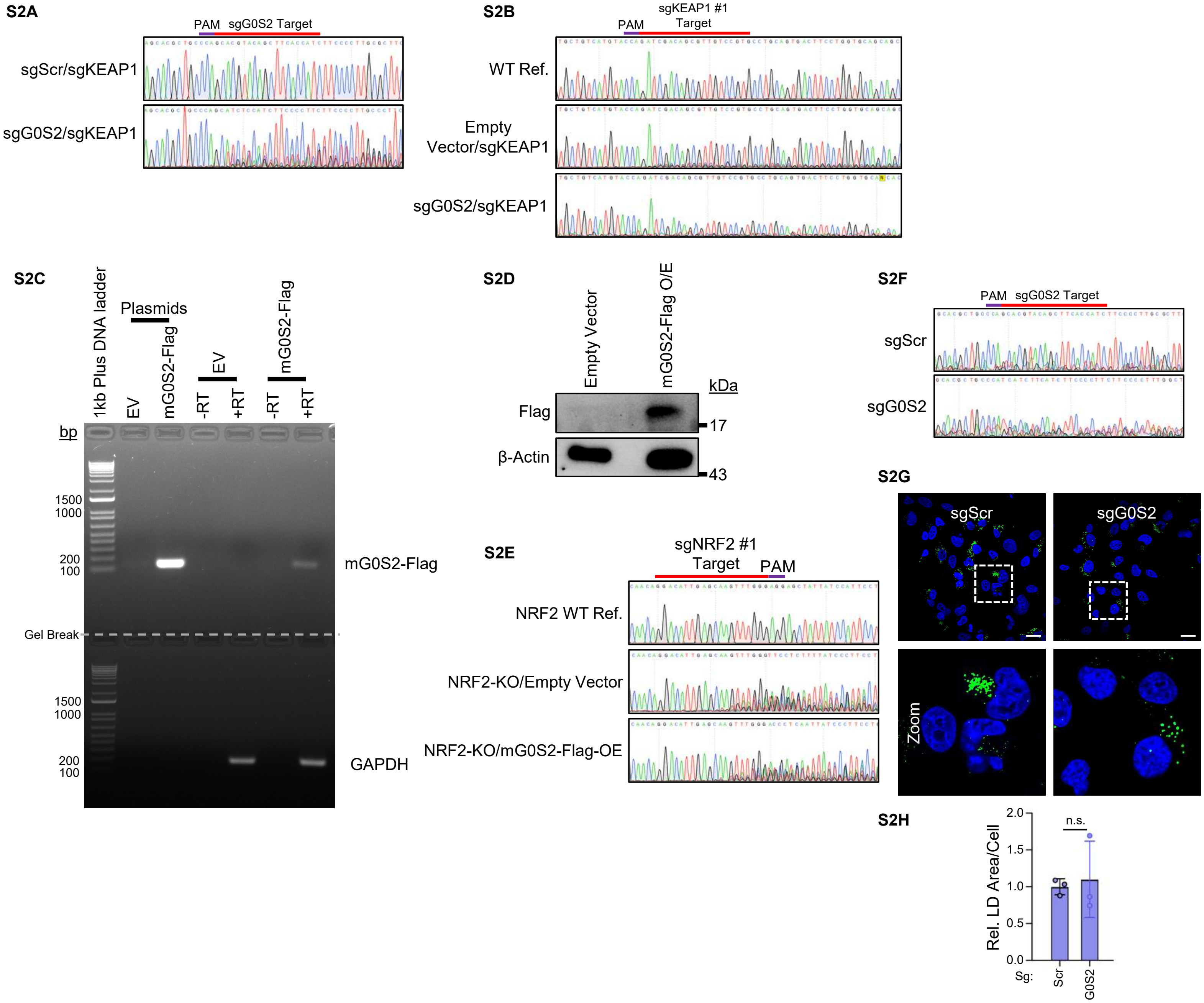

Supplemental Figure 2. Validation of genetic G0S2 manipulations in HEK-TtH cell lines and sgG0S2 microscopy.

(A) Chromatogram of genomic DNA sequencing of the G0S2 gene in HEK-TtH cells transduced with sgKEAP1 #1 plus either sgG0S2 or sgScr (mixed populations). sgG0S2 targets exon 2 (first translated coding exon) at sequence 5’- GCACGTACAGCTTCACCATC-3’.

(B) Chromatogram of genomic DNA sequencing of the KEAP1 gene in HEK-TtH cells transduced with sgKEAP1 # plus either sgG0S2 or sgScr (mixed populations). sgKEAP1 #1 targets exon 2 at sequence 5’- CACGGACAACGCTGTCGATC-3’.

(C) PCR amplification of mG0S2-Flag either directly from plasmid DNA or from cDNA generated from HEK-TtH cells stably infected with empty vector or virus encoding mG0S2-Flag.

(D) Immunoblot for Flag in NRF2-KO cells transduced with empty vector or mG0S2-Flag.

(E) Chromatogram of genomic DNA sequencing of the NRF2 gene in HEK-TtH cells transduced with sgNRF2 #1 and overexpressing empty vector or mG0S2-Flag (mixed populations). sgNRF2 #1 targets exon 4 at sequence 5’- GGACATTGAGCAAGTTTGGG-3’.

(F) Chromatogram of genomic DNA sequencing of the G0S2 gene in HEK-TtH cells transduced with either sgG0S2 or sgScr (mixed populations). sgG0S2 targets exon 2 (first translated coding exon) at sequence 5’- GCACGTACAGCTTCACCATC-3’.

(G) Representative images of LDs in sgG0S2 HEK-TtH cells taken with 63x objective. Scale bars represent 20µm.

(H) Quantification of LD area per cell from (S2G). n = 3 biological replicates. Student’s t-Test (one-tailed, unpaired with equal variance). Mean ± SD.

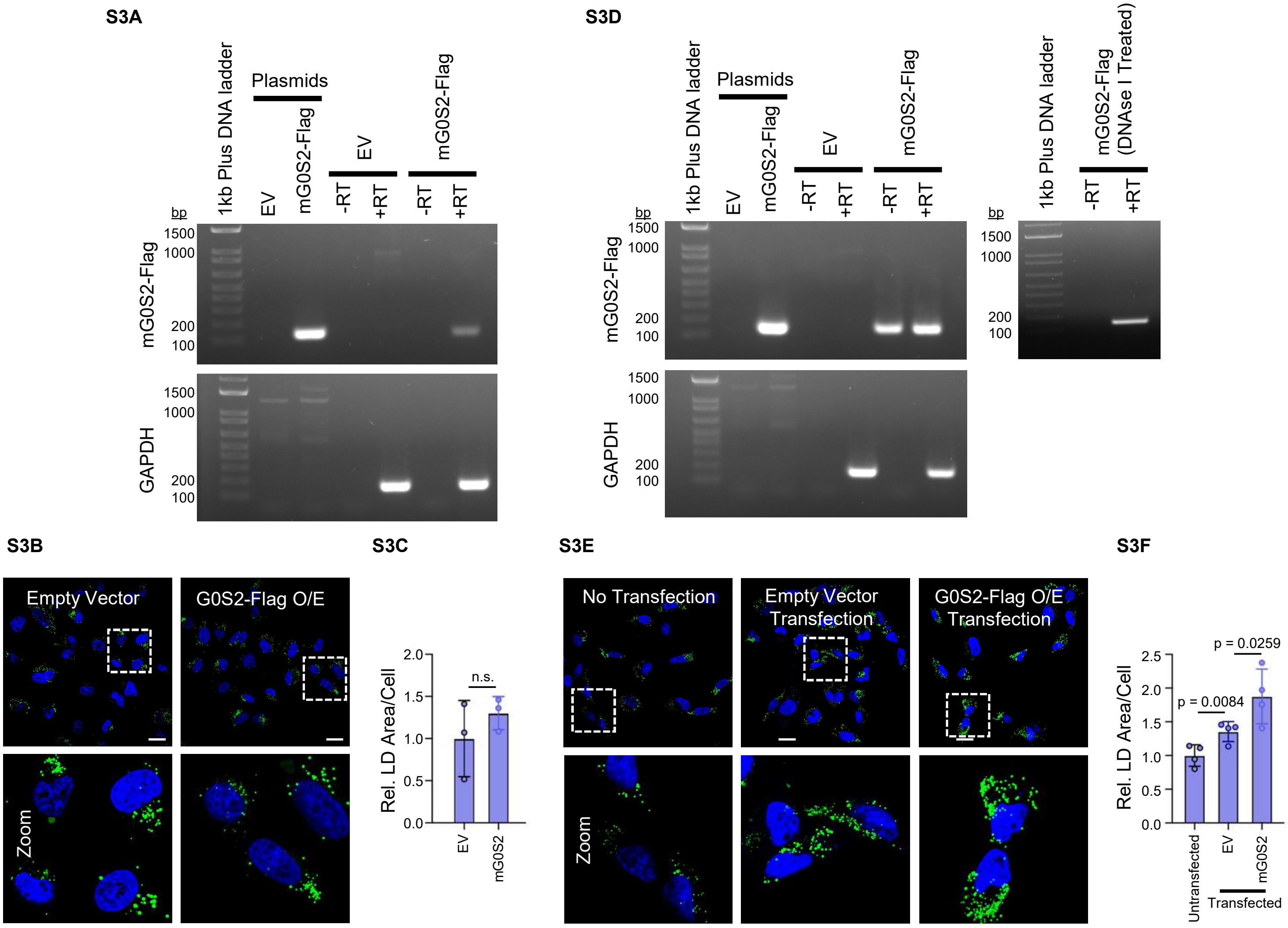

Supplemental Figure 3. mG0S2-Flag overexpression in HEK-TtH cells influence on LD content.

(A) PCR amplification of mG0S2-Flag either directly from plasmid DNA or from cDNA generated from HEK-TtH cells stably infected with empty vector or virus encoding mG0S2-Flag.

(B) Representative images of LDs in mG0S2-Flag overexpressing HEK-TtH cells taken with 63x objective. Scale bars represent 20μm.

(C) Quantification of LD area per cell from (S3B). n = 3 biological replicates. Student's t-Test (one-tailed, unpaired with equal variance). Mean ± SD.

(D) PCR amplification of mG0S2-Flag either directly from plasmid DNA or from cDNA generated from HEK-TtH cells transfected with empty vector or plasmid encoding mG0S2-Flag. RNA samples of top right panel were DNAse I treated and purified prior to reverse transcriptase reaction.

(E) Representative images of LDs in mG0S2-Flag transfected HEK-TtH cells taken with 63x objective. Scale bars represent 20μm.

(F) Quantification of LD area per cell from (S3E). n = 4 biological replicates. Student's t-Test (one-tailed, unpaired with equal variance). Mean ± SD.

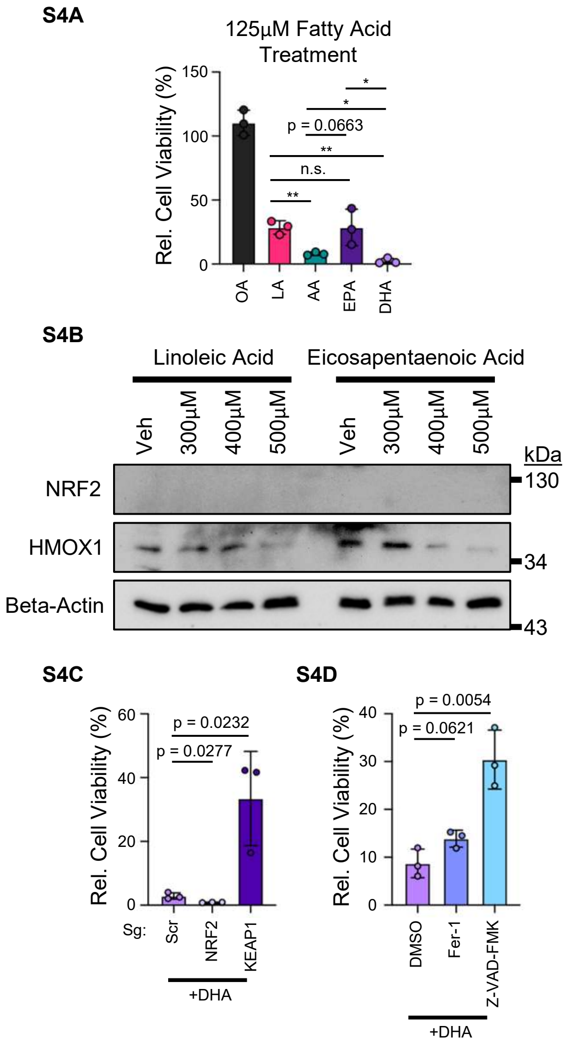

Supplemental Figure 4. Additional fatty acid treatment data.

(A) Quantification of cell viability (CellTiter-Glo) of HEK-TtH cells following treatment with the indicated fatty acids (125µM). n=3 biological replicates of each. Mean ± SD. OA treated comparison to all other fatty acid treatments is statistically significant with $p \leq 0.0012$. (not significant = n.s., p<0.05 = *, p<0.01 = **, p<0.001 = ***, p<0.0001 = ****).

(B) Immunoblot of NRF2 and HMOX1 following 4-hour of treatment with the indicated doses of linoleic acid (LA) or Eicosapentaenoic acid (EPA) conjugated to fatty acid-free BSA.

(C) Quantification of cell viability (CellTiter-Glo) relative to vehicle control of sgNRF2 and sgKEAP1 HEK-TtH cells treated for 24 hours with 200µM docosahexaenoic acid (DHA) conjugated to fatty acid-free BSA. n=3 biological replicates of each. Mean ± SD.

(D) Quantification of cell viability (CellTiter-Glo) relative to vehicle control of HEK-TtH cells treated for 24 hours with 200µM docosahexaenoic acid (DHA) conjugated to fatty acid-free BSA and co-treated with 10µM Ferrostatin-1 (Fer-1) or 10µM Z-VAK-FMK. n = 3 biological replicates of each. Mean ± SD.

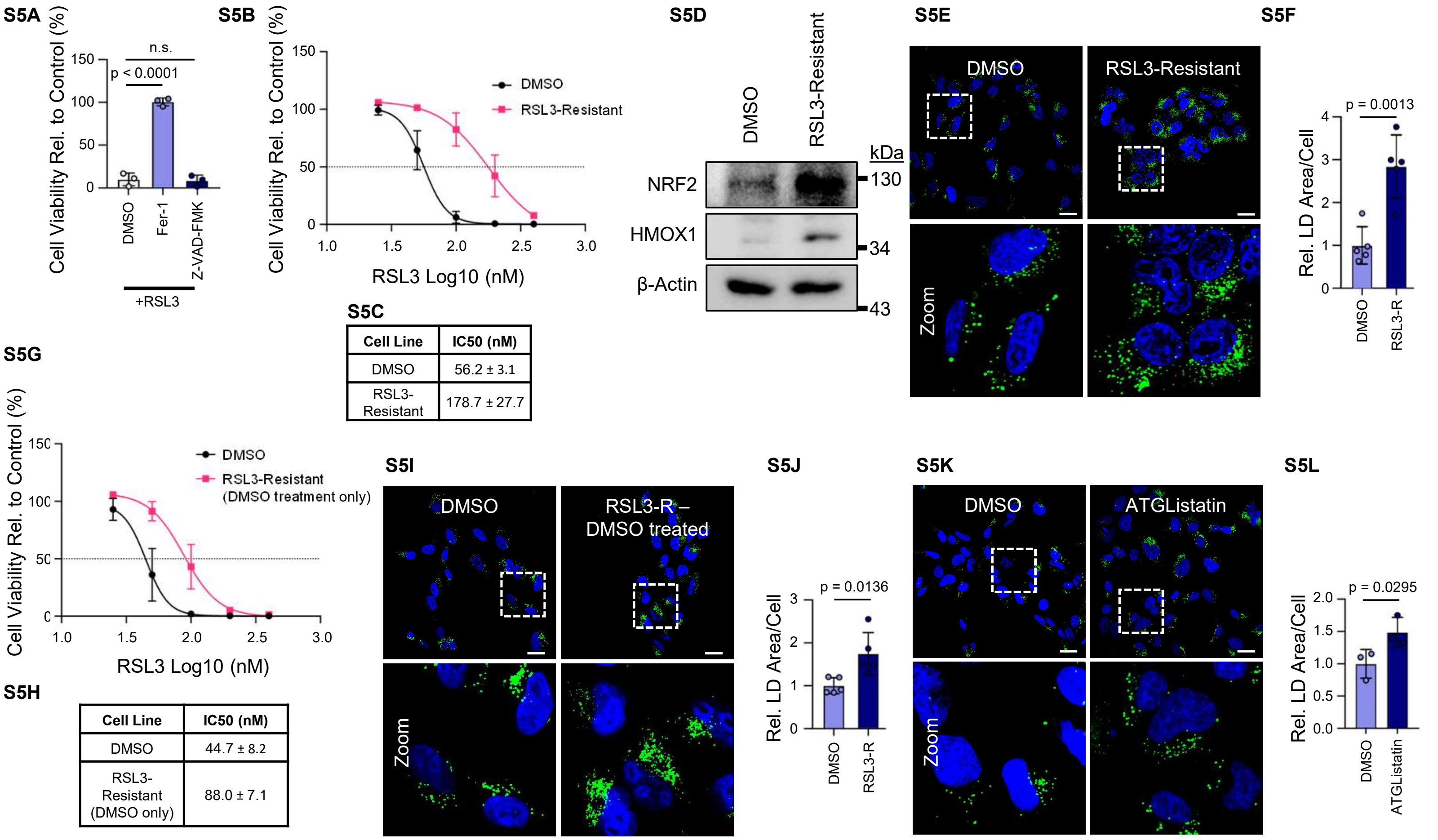

S5C

Cell Line	IC50 (nM)
DMSO	56.2 ± 3.1
RSL3-Resistant	178.7 ± 27.7

S5H

Cell Line	IC50 (nM)
DMSO	44.7 ± 8.2
RSL3-Resistant (DMSO only)	88.0 ± 7.1

Supplemental Figure 5. Validation of reagents and ability of cells to maintain RSL3-resistance.

(A) Quantification of cell viability (CellTiter-Glo) relative to vehicle control of HEK-TtH cells treated with 100nM RSL3 and co-treated with 10μM Ferrostatin-1 (Fer-1) or 10μM Z-VAK-FMK for 24 hours. n = 3 biological replicates of each. Mean ± SD.

(B) Quantification of cell viability (CellTiter-Glo) RSL3 resistant HEK-TtH cells continuously maintained on 100nM RSL3 or control HEK-TtH cells maintained with an equivalent volume of DMSO and then treated for 24 hours with gradient of RSL3. Horizontal dotted line represents half maximal inhibitory concentration (IC_{50}). n = 4 biological replicates. Mean ± SD.

(C) IC_{50} and standard error calculated from (B) for RSL3-resistant cells and equivalent DMSO treated cells.

(D) Immunoblot for NRF2 and HMOX1 in RSL3-resistant HEK-TtH cells.

(E) Representative images of LDs in RSL3-resistant HEK-TtH and control cells taken with 63x objective. Scale bars represent 20μm.

(F) Quantification of LD area per cell from (E). n = 5 biological replicates. Mean ± SD.

(G) Quantification of cell viability (CellTiter-Glo) relative to vehicle control of RSL3-resistant HEK-TtH cells that have been removed from RSL3 for at least 6 days and continuously treated with DMSO. Cells treated for 24 hours with gradient of RSL3. Horizontal dotted line represents half maximal inhibitory concentration (IC_{50}). n = 4 biological replicates. Mean ± SD.

(H) IC_{50} and standard error calculated from (G) for RSL3-resistant cells removed from RSL3 for at least 6 days and equivalent DMSO treated cells.

(I) Representative images of LDs in RSL3-resistant HEK-TtH cells following RSL3 removed for at least 6 days and control cells continuously treated with DMSO taken with 63x objective. Scale bars represent 20μm.

(J) Quantification of LD area per cell from (I). n = 5 biological replicates. Mean ± SD.

(K) Representative images of LDs in HEK-TtH cells treated with 10μM ATGListatin or DMSO for 24hrs taken with 63x objective. Scale bars represent 20μm.

(L) Quantification of LD area per cell from (K). n = 3 biological replicates. Student's t-Test (one-tailed, unpaired with equal variance). Mean ± SD.

Table S1. sgTarget Sequences (5'-3')	Source
sgScr - GTATTACTGATATTGGTGGG	Sessions et al., 2022
sgNFE2L2 (NRF2) sg1 - GGACATTGAGCAAGTTTGGG	This study
sgNFE2L2 (NRF2) sg2 - GCCACAGTCAACACAGATTT	This study
sgKEAP1 sg1 - CACGGACAACGCTGTCGATC	This study
sgKEAP1 sg2 - CTACCTGGTCAAGATCTTCG	This study
sgG0S2 - GCACGTACAGCTTCACCATC	This study

Table S2. PCR and Sequencing Oligonucleotides (5'-3')	Source
G0S2 sg PCR F - CTCCTCCTGCAGGTCATTCC	This study
G0S2 sg PCR R - CAGTTCCTAGGAGGCGTGC	This study
G0S2 sg R seq oligo - CCTTTCTCCTGCAGGGCTTGC	This study
KEAP1 sg1 PCR F - CCTCCCAGCATGGCAACC	This study
KEAP1 sg1 PCR R - CAACTCCACACAGCCAATC	This study
KEAP1 sg1 seq oligo - GCAGGTCAAGTACCAGGATG	This study
KEAP1 sg2 PCR F - GGTCCTGCTTGGTGAGGTGTG	This study
KEAP1 sg2 PCR R - GCCTCAGGAAGAATACCCGG	This study
KEAP1 sg2 seq oligo - GACGACCTGAACGTGCGCTG	This study
NFE2L2 (NRF2) sg1 PCR F - CTGGTTAGTAAGTAGAGAGAC	This study
NFE2L2 (NRF2) sg1 PCR R - GATGCCACACTGGGACTTG	This study
NFE2L2 (NRF2) seq oligo - CTTCGGCTACGTTTCAGTC	This study
mG0S2 F RT-PCR - CTTGTGAACAGCAGTCCCTC	This study
Flag R RT-PCR - CGTCGTCATCCTTGTAATC	This study
GAPDH F RT-PCR - AACGTGTCAGTGGTGGACCT	Arras et al., 2021
GAPDH R RT-PCR - TCGCTGTTGAAGTCAGAGGA	Arras et al., 2021

Table S3. Oligonucleotides (5'-3')	Source
ACSL4 F - CTTGCTTTACCTATGGCTGC	This study
ACSL4 R - CTTCAGTACAGTACAGTCTC	This study
ATGL F - ATGGTGGCATTTCAGACAACC	Jin et al., 2018
ATGL R - CGGACAGATGTCACTCTCGC	Jin et al., 2018
CD36 F - AAGCCAGGTATTGCAGTTCTTT	Lee et al., 2013
CD36 R - GCATTTGCTGATGTCTAGCACA	Lee et al., 2013
CGI-58 F - GGTGGATTCTTGGCTGCTG	This study
CGI-58 R - GGCTCTGATCCAAACTGG	This study
CPT1A F - GATTTTGCTGTCGGTCTTGG	Cifre, Palou, and Oliver, 2020
CPT1A R - CTCTTGCTGCCTGAATGTGA	Cifre, Palou, and Oliver, 2020
FASN F - CTTCCGAGATTCCATCCTACGC	Li et al., 2014
FASN R - TGGCAGTCAGGCTCACAAACG	Li et al., 2014

FSP27 F - ATTGATGTGGCCCGTGTAACG	Jin et al., 2018
FSP27 R - CAGCAGTGCAGATCATAGGAAA	Jin et al., 2018
G0S2 F - CTGCCGAGAGGAGGAGAAC	This study
G0S2 R - CCGTTTCCATCTCGGCTC	This study
GAPDH F - AACGTGTCAGTGGTGGACCT	Arras et al., 2021
GAPDH R - TCGCTGTTGAAGTCAGAGGA	Arras et al., 2021
GCLM F - GGTCAGGGAGTTTCCAGATG	This study
GCLM R - GTGCAACTCCAAGGACTGAACAG	This study
HILPDA F - CTGGTGCTTAGTAACCGAC	This study
HILPDA R - CAACACATGCTTCATGGC	This study
NQO1 F - GGACATCACAGGTAAACTG	This study
NQO1 R - GCAGGGACTCCAAACCACTG	This study
PLIN1 F - GACACCAGCAAGCCCAGAAG	This study
PLIN1 R - GCCACTGAGGCACCCCACTG	This study
PLIN2 F - CTAGACAGGATTGAGGAGAGAC	This study
PLIN2 R - CACAGTAGTCGTCACAGCATC	This study
PLIN3 F - GCCGATCCTCTCCAAGCTGGAG	This study
PLIN3 R - GACACCTTAGACGACACAAG	This study
PLIN4 F - CTCTGGCCCAGCTCCAGGAC	This study
PLIN4 R - CAGCTGCCGGAGAAGGCCGC	This study
PLIN5 F - CTGGTGGATCACTTCCTGCC	This study
PLIN5 R - CTCTGATCCTCCACCGAACC	This study
